# Retraction: Sodium thiosulfate through preserving mitochondrial dynamics ameliorates oxidative stress induced renal apoptosis and ferroptosis in 5/6 nephrectomized rats with chronic kidney diseases

**DOI:** 10.1371/journal.pone.0295162

**Published:** 2023-11-28

**Authors:** The PLOS ONE Editors

After this article [1] was published, concerns were raised about some of the panels in Figs 5–8. Specifically:

- In Fig 5A, the two H&E stain Sham panels are duplicated in the cortex Sham panels in Fig 4A in [2].
- In Fig 5B, the two Masson stain Sham panels are duplicated in the cortex Sham panels in Fig 4B in [2].
- In Fig 5C, the two H&E stain Sham panels are duplicated in the medulla Sham panels in Fig 4A in [2].
- In Fig 5D, the two Masson stain Sham panels are duplicated in the medulla Sham panels in Fig 4B in [2].
- The β-actin 42kDa panel in Fig 6A appears similar to the β-actin 42kDa panel in Fig 8A.
- In Fig 6C, the two Sham panels are duplicated in the Sham panels in Fig 7C when rotated 180°, the cortex Sham panels in Fig 5B in [2], and the cortex Sham panels in Fig 6B in [2] when rotated 180°.
- In Fig 6D, the two Sham panels are duplicated in the medulla Sham panels in Fig 5B in [2].
- In Fig 6D, the two CKD panels are duplicated in the CKD panels in Fig 7D.
- In Fig 7D, the two Sham panels are duplicated in the medulla Sham panels in Fig 6B in [2].
- In Fig 8E, the two Sham panels appear similar to the Sham panels in Fig 7B in [2].
- In Fig 8F, the two Sham panels appear similar to the Sham panels in Fig 7C in [2].

During editorial follow-up on these issues, the corresponding author stated that the eight Sham panels in Figs 5A-D, the four Sham panels in Figs 6C-D, the two CKD panels in Fig 6D, and the four Sham panels in Figs 7C-D in [1] were included in error.

In light of the extent of the above issues with Figs 5–8 that question the reliability of these data, the *PLOS ONE* Editors retract this article.

CTC agreed with the retraction. YHC, CAY, CCY, and SPH either did not respond directly or could not be reached.

Owing to the concerns about similarities with previously published content [2], published in 2021 by S. Karger AG [2], which is not offered under a CC BY license, the eight Sham panels in Figs 5A-D, the four Sham panels in Figs 6C-D, the four Sham panels in Figs 7C-D, and the four Sham panels in Figs 8E-F are excluded from this article’s [1] license. At the time of retraction, the article [1] was republished to note these exclusions in the legends of Figs 5–8 and the article’s copyright statement.
